# The effects of home confinement on pediatric fractures during the COVID 19 outbreak

**DOI:** 10.1186/s13052-021-01092-7

**Published:** 2021-06-30

**Authors:** Jun Li, Xiaowei Yuan, Yinqiang Cao, Tao Liu, Pan Gou, Xiang Li, Ming Li, Xing Liu

**Affiliations:** 1 Department I of Orthopedic of Children’s Hospital of Chongqing Medical University, No. 136 of Zhong Shan Er Lu, Chongqing, 400014 China; 2 Ministry of Education Key Laboratory of Child Development and Disorders, No. 136 of Zhong Shan Er Lu, Chongqing, 400014 China; 3 National Clinical Research Center for Child Health and Disorders, No. 136 of Zhong Shan Er Lu, Chongqing, 400014 China; 4 China International Science and Technology Cooperation base of Child development and Critical Disorders Chongqing Key Laboratory of Pediatrics, Children’s Hospital of Chongqing Medical University, No. 136 of Zhong Shan Er Lu, Chongqing, 400014 China

**Keywords:** Pediatric fractures, Epidemiology, Home confinement, COVID-19

## Abstract

**Objective:**

To control the transmission of coronavirus disease 2019 (COVID-19), the Chinese government encouraged people to stay at home. This study aimed to evaluate the effects of home confinement on the occurrence of fractures among children.

**Study design:**

We retrospectively reviewed children admitted to Children’s Hospital of Chongqing Medical University, for traumatic injury from January 24 to March 10, 2020, and the same time period in 2017, 2018 and 2019. At the same time, children with fracture were screened out and the date for the past 4 years was compared in terms of etiology, location of fracture, sex and age to evaluate the effects of home confinement on the epidemiology of pediatric fractures during the COVID-19 outbreak.

**Results:**

There were 6066 fractures in5,346 patients in 2017–2019, and 1034 fractures in 862 patients in 2020; the number of patients in all years reached a peak at the age of 2 to 4 years. The patients were slightly younger in 2020 than in 2017–2019 (t = 9.953, 95% CI: 0.846–1.262), and the proportion of boys in 2017–2019 is higher than in 2020 (X^2^ = 6.944, *P* = 0.008). Home confinement and traffic restriction resulted in a reduction in traffic accidents-associated fractures among children (X^2^ = 16.399, *P* < 0.001).

**Conclusion:**

Home confinement lead to the significant reduction in the number of pediatric fractures, especially in male children, but the number of patients under 4 years old was still considerable, and the proportion of younger patients even increased. Therefore, the perspective of children should not be relaxed during home isolation.

## Introduction

Traumatic injury contributes to a vast majority of mortality and physical disability across the world, with nearly 1 in 4 children getting injured annually [1]. Almost 25% of pediatric trauma cases in emergency departments are treated for fractures. Pediatric fracture is a significant public health issue [2]; it leads to a variable spectrum of complications including epiphyseal injury, joint stiffness, traumatic arthritis, ischemic osteonecrosis, and osteofascial compartment syndrome, which brings both physical pain and potential limb deformities to the children.

Many factors affect the incidence of fractures in children, including age, sex, traffic, physical activity, child behavior, and parents’ awareness, etc. [3]. In order to curb the transmission of coronavirus disease 2019 (COVID-19), the Chinese government has ordered a nation-wide school closure as an emergency measure to prevent the spreading of the infection. Public activities are also prohibited. The Ministry of Education estimated that more than 220 million children and adolescents are confined to their homes; this includes 180 million primary and secondary students and 40 million preschool children [4]. Home confinement resulted in a reduction in children’s activity and parents had more time to look after their children; at the same time, a large number of vehicles are out of service, contributing to a dramatic reduction in traffic accidents [5]. These factors are closely related to the incidence of fractures in children [3].

The primary objective of this study was to determine the effect of home confinement on the epidemiology of pediatric fractures. A secondary objective was to facilitate the understanding of risk factors for fractures in children, and the proposal of effective protective measures.

## Methods

### Setting

The study used data from Children’s Hospital of Chongqing Medical University, which is the largest Children hospital in southwest China. With the joint efforts of the Chinese government and the people, the COVID-19 was brought under control in early March; after that, restrictions on residents’ travel was eased. To evaluate the effects of home confinement on the occurrence of fractures among children, the clinical and radiographic date of all admitted children under 16-years old with traumatic injury between Jan 24 and Mar 10, 2020, and the same period in 2017, 2018 and 2019 was extracted from a hospital information system. This study was exempted from ethical approval because patient data were completely de-identified.

### Study population

The study population included all admitted children under16-years old with traumatic injurybetween Jan 24 and Mar 10, 2020, and the same period in 2017, 2018 and 2019. Fractures were identified based on 5-digit ICD-9 codes. All X-rays were reviewed by a radiologist to ensure all non-fracture or soft-tissue injuries were excluded. Of them, the patients with fractures were selected for retrospective analysis. The population was stratified into four age groups. These were infants (0–1 years), pre-school children (2–4 years), school children (5–11 years) and adolescents (12–16 years).

### Statistical analysis

The data was collected using Microsoft Excel sheets and statistical analysis were performed using SPSS 25. Frequency (percentage) was reported. Differences were assessed with Pearson χ^2^ for categorical variables. The critical *P*-value for significance was set at 0.05.

## Results

**Fig. 1:**
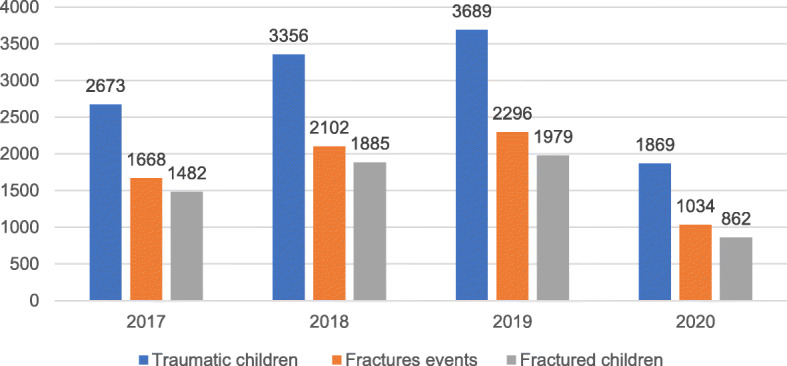
Case summary chart

A total of 11,587 children with traumatic injury, and 6208 patients with 7100 fractures events were enrolled in the study. In 2017–2019, 9718 children experienced trauma, of which 5346 children had 6066 fractures events, whereas 862 of 1869 traumatic children had 1034 fractures events in 2020(Fig. 1). The fracture rate among children with trauma was 55.01% in 2017–2019, significantly higher than 46.12% in 2020 (X2 = 49.813, *P* < 0.001). Meanwhile, the fracture rate in the males was higher in 2017–2019 than in 2020(Table 1).

**Table 1:** Fractures ratios in different genders

FRACTURES RATIOS GENDER	2017	2018	2019	2020	X^**2**^	***P***
**MALE**	937/1678(55.84%)	1175/2080(54.50%)	1258/2219(56.69%)	503/1108(45.40%)	45.814	< 0.001
**FEMALE**	545/995(54.77%)	710/1276(55.64%)	721/1470(49.05%)	359/761(47.17%)	22.063	< 0.001
**TOTAL**	1482/2673(55.44%)	1885/3356(56.17%)	1979/3689(53.65%)	862/1869(46.12%)	54.584	< 0.001

The mean age of patients with fractures was 5.64 years (SD 3.937) in 2017–2019, higher than 4.58 years (SD 3.445) in 2020 (t = 9.953, 95% CI: 0.846–1.262). In 2017–2019, 3370 male and 1976 female children had fractures, with a sex ratio (Male/Female) of 1.71. Fractures occurred more commonly in boys than in girls in 2017–2019; however, in 2020, the difference decreased in the number of male and female patients. There were 503 male cases and 359 female cases, with a sex ratio (Male/Female) of 1.40. The proportion of boys in 2017–2019 is higher than in 2020 (X2 = 6.944, *P* = 0.008).

**Fig. 2:**
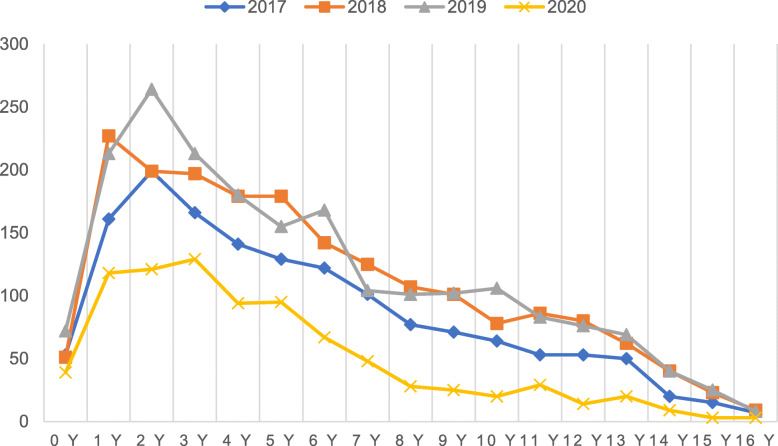
Overall number of cases curves

**Fig. 3:**
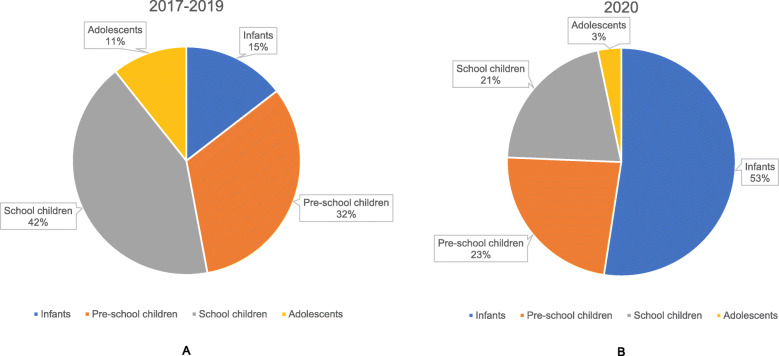
Pie chart of age groups in 2017–2019 (**A**) and 2020 (**B**)

The age-stratified number of patients with fractures was illustrated in Fig. 2, which showed a peak at the age of 2 to 4 years in all years. In 2017–2019, the age group with the largest number of patients was school children (5–11 years) with, 2260 cases (42.27%). In 2020, pre-school children (2–4 years) ranked top of the number of patients with 344 cases (39.90%) (Fig. 3). The adolescents (12–16 years) had the lowest number of patients in both years and had the most significant sex ratio, 3.84 in 2017–2019, 3.08 in 2020. Although the sex ratios of each age group are different between 2017 and 2019 and 2020, there was no significant difference in all groups expect school children group (Table 2). The proportion of adolescents in 2020 was significantly reduced from 2017 to 2019 (X2 = 20.614, *P* < 0.001) Table 3).

**Table 2:** Characteristics of sex ratios in different age groups

SEX RATIOS (MALE/FEMALE) AGE GROUPS	2017	2018	2019	2020	X^**2**^	***P***
**INFANTS**	1.22	1.48	1.71	1.37	3.425	0.331
**PRE-SCHOOL CHILDREN**	1.29	1.37	1.46	1.32	1.204	0.752
**SCHOOL CHILDREN**	2.05	1.65	1.72	1.35	9.245	0.026
**ADOLESCENTS**	4.57	3.73	3.54	3.08	1.323	0.724

**Table 3:** Characteristics of Proportion of patients in different age groups

PROPORTION OF PATIENTS AGE GROUPS	2017	2018	2019	2020	X^**2**^	***P***
**INFANTS**	214(14.44%)	278(14.75%)	285(14.40%)	157(18.21%)	7.967	0.047
**PRE-SCHOOL CHILDREN**	506(34.14%)	575(30.50%)	657(33.20%)	344(39.91%)	23.818	< 0.001
**SCHOOL CHILDREN**	617(41.63%)	824(43.71%)	819(41.38%)	312(36.19%)	13.813	0.003
**ADOLESCENTS**	145(9.78%)	208(11.03%)	218(11.02%)	49(5.68%)	22.449	< 0.001

There were 7100 fractures in total, and 769 patients (12.39%) presented with multiple fractures, 623 patients in 2017–2019, 146 patients in 2020. In terms of fracture sites, the most type was humeral fractures in all years, 1675 cases (27.61%) in 2017–2019, and 381 cases (36.84%) in 2020. The proportion of skull fracture and humeral fracture in 2020 was higher than in 2017–2019 (X2 = 69.999, X2 = 36.617, *P* < 0.001) (Table 4). A total of 556 fractures required surgery in 2017–2019, which decreased to 81 cases in 2020; however, there was no significant difference in proportion of surgical fractures in 2020 and 2017–2019 (Table 5).

**Table 4:** Characteristics of Proportion of patients in different fracture sites

PROPORTION OF PATIENTS FRACTURE SITES	2017	2018	2019	2020	X^**2**^	***P***
**HUMERUS**	457(27.40%)	575(27.35%)	643(28.01%)	381(36.85%)	36.895	< 0.001
**RADIUS**	227(13.61%)	333(15.84%)	275(11.98%)	119(11.51%)	18.007	< 0.001
**ULNA**	216(12.95%)	311(14.80%)	272(11.85%)	133(12.86%)	8.541	0.036
**TIBIA**	211(12.65%)	241(11.47%)	208(9.06%)	52(5.03%)	48.548	< 0.001
**CLAVICLE**	139(8.33%)	128(6.09%)	183(7.97%)	86(8.32%)	9.345	0.025
**PHALANX**	45(2.70%)	58(2.76%)	67(2.92%)	45(4.35%)	7.403	0.006
**FEMUR**	128(7.67%)	178(8.47%)	192(8.36%)	32(3.09%)	34.433	< 0.001
**SKULL**	14(0.84%)	30(1.43%)	33(1.44%)	52(5.03%)	72.384	< 0.001
**FIBULA**	73(4.38%)	72(3.43%)	78(3.40%)	28(2.71%)	5.740	0.125
**OTHERS**	115(6.89%)	112(5.33%)	254(11.06%)	106(10.25%)	53.329	< 0.001

**Table 5:** Operation ratios in different Fracture sites

OPERATION RATIOS FRACTURE SITES	2017	2018	2019	2020	X^**2**^	***P***
**HUMERUS**	76/457(16.63%)	103/575(17.91%)	90/643(14.00%)	46/381(12.07%)	7.548	0.056
**FEMUR**	12/128(9.38%)	30/178(16.85%)	18/192(9.38%)	11/32(34.38%)	18.434	< 0.001
**TIBIA**	24/211(11.37%)	32/241(13.27%)	14/208(6.73%)	3/52(5.77%)	6.626	0.085
**RADIUS**	9/227(3.96%)	16/333(4.80%)	6/275(2.18%)	4/119(3.36%)	3.025	0.388
**PHALANX**	7/45(15.56%)	10/58(17.24%)	8/67(11.94%)	4/45(8.89%)	1.818	0.611
**ULNA**	7/216(3.24%)	7/311(2.25%)	3/272(1.10%)	4/133(3.01%)	2.934	0.402
**OTHERS**	17/384(4.43%)	39/406(9.61%)	34/639(5.32%)	9/272(3.31%)	15.395	0.002
**TOTAL**	152/1668(9.11%)	237/2102(11.27%)	167/2296(7.27%)	81/1034(7.83%)	23.442	< 0.001

The most common injury mechanism leading to fractures in children was a fall on the same plane in all years, 4090 cases (76.51%) in 2017–2019, 707 cases (82.02%) in 2020. Home confinement and traffic restriction contributed to the reduction in traffic accidents-associated fractures among children (X2 = 16.399, *P* < 0.001) Table 6).

**Table 6:** Characteristics of Proportion of patients in different etiologies

PROPORTION OF PATIENTS ETIOLOGY	2017	2018	2019	2020	X^**2**^	***P***
**FALL ON THE SAME PLANE**	1148(77.46%)	1394(73.95%)	1548(78.22%)	707(82.02%)	23.933	< 0.001
**CRUSH INJURY**	58(3.91%)	93(4.93%)	102(5.15%)	33(3.83%)	4.616	0.202
**TRAFFIC ACCIDENTS**	82(5.53%)	86(4.56%)	78(3.94%)	14(1.62%)	21.761	< 0.001
**HIGH FALLING INJURY (> 2 M)**	39(2.63%)	48(2.55%)	50(2.53%)	9(1.04%)	7.454	0.006
**CRASH INJURY OF A HEAVY OBJECT**	15(1.01%)	17(0.90%)	17(0.86%)	11(1.28%)	1.217	0.749
**OTHERS**	140(9.45%)	247(13.10%)	184(9.30%)	88(10.21%)	18.203	< 0.001

## Discussion

Fracture in children is a significant public health issue and a frequent cause of emergency room visits; its diagnosis and treatment are distinctive. WHO declared the COVID-19 outbreak, caused by severe acute respiratory syndrome coronavirus 2, to be a pandemic on March 11, 2020. In order to curb spread of COVID-19, the Chinese government decided to close the school and encouraged people to stay at home [5]. Stay-at-home orders resulted in a significant reduction in the number of admitted children with fractures compared to the same period in 2017–2019. There were 5346 patients with 6066 fractures that happened in 2017–2019 and 862 patients with 1034 fractures happened in 2020. The reduction in the number of children with fractures might be attributable to the following reasons: 1. Sport and game-playing contributed to a large proportion of fracture events in children. Home confinement reduced the opportunities for physical activity among children, particularly for children living in small apartments in urban areas, which ultimately reduced the risk of fractures [6, 7]. 2.During the epidemic, the children stayed with their parents all day; parental supervision of children was strengthened, which could prevent children from injury in time [8]. 3. Chinese government enforced the restriction of public transport, leading to a decline in traffic accidents, which subsequently reduced the accidents-associated fractures in children.

The fractures ratio in this study was higher than previous researches [2], which might be due to the fact that many children with low-energy trauma that did not cause fractures were treated in primary-level hospitals. Most fractures in older children occurred outdoors; home confinement could reduce the out-door activities, which subsequently resulted in a dramatic decline in the fracture ratio among school children and adolescents in 2020. During home isolation, the number of patients requiring operation treatment was decreased [9].

The patients in 2020 were younger than those in 2017–2019; the main reason was that the activity space of younger children, such as infants and pre-school children, is smaller than that of older children, and the daily activities of younger children are not significantly reduced. The incidence of fractures due to physical activity and sports increases with age; there was a greater impact on the incidence of older children’s fractures than younger children, leading to the old patients significantly reduced in 2020 and the mean age declined.

Previous studies have shown that male children were more prone to fractures than female [10]; however, our study indicated only marginal difference, 58.35% in boys compared to 41.65% in girls in 2020. Most scholars believed the underlying cause reason of more fractures in boys is due to their relatively higher-level activity and their interests of taking risks, which may easily lead to the onset of fractures [11–13]. We supposed that the restrictions on boys’ activities under home quarantine were more evident than that of girls’, leading to lower sex ratio (males to females) and lower fractures ratio in boys in 2020.

The number of patients in both 2017–2019 and 2020 reached the peak at the age of 2 to 4 years, and that number in pre-school children was even higher than in school children in 2020. It is mainly due to the intense curiosity of pre-school children, who have the sense of self-action, but the poor ability of risk assessment and self-protection. Therefore, parents should take good care of children between 2 and 4 years old, even during the home isolation. Parents’ awareness of preventing children from injury prevention have a positive effect on lowering the risk of children injuries at home [3]. The age distribution of fractures of this study was different to patterns observed in Europe/North American which usually peaking during puberty [10], the main reason was that most teenagers go to adult hospitals for treatment and we couldn’t get the data of these patients.

Home isolation seems to not impact the severity and site of fractures, and the surgery requirement. The upper limb fractures were still the most common type in children, which is consistent with most published literature [1]. Home isolation narrowed the child’s activity space, which made the child more vulnerable to head injuries. Resulted in the proportion of skull fracture in 2020 was higher than in 2017–2019 (X^2^ = 69.999, *P* < 0.001), The incidence of traffic accidents-associated fractures in children was reduced due to the traffic restriction, and falling on the same plane was still the most common cause. Even during home isolation, there were still a large number of children who suffered fractures as a result of falls, and the proportion was even higher than in previous years. Therefore, the supervision of children even during home isolation should not be relaxed.

The social distancing and stay-at-home orders, it leaded to a significant decrease in the amount of activity of the children. The risk of childhood obesity is significantly increased due to lack of activity and obesity in children way lead to decreased bone density [14, 15]. International physical activity guidelines for children recommend that children should participate in at least 60 min of moderate or vigorous physical activity every day [16]. Although physical activity makes children at high risk of fractures; however, in the long run increased physical activity is associated with decreased fracture risk, probably in part due to beneficial gains in areal bone mineral density (aBMD) and muscle strength [1, 17]. We recommend appropriate exercise for children while ensuring safety during the home confinement.

According to researches, pediatric fractures had apparent seasonal rhythm, and the incidence is higher in summer and autumn [2]. It was summer when home confinement end, and the school reopened, children cannot wait to play and exercise after a long period of activity restrictions. It could be anticipated that once the home confinement ends, the incidence of pediatric fractures may be significantly increased. Home isolation leaded to a decrease in the incidence of fractures in children, but the number of children with fractures was still considerable, teachers and parents are required to strengthen the care of children either at home or in school during home isolation or after. At the same time, the pediatricians should be prepared to admit children with fractures.

### Limitations

The main limitation of the study was mainly due to the characteristic of a single-center retrospective study, with a limited sample size. Bias during the research seemed to be unavoidable. We cannot predict the impact of extended home isolation time on children’s fracture epidemiology due to the short period for research data. The epidemiology of fracture in children was influenced by region, race and season; the results of this study might not be generalized to the children all over the world, but still could provide valuable references for the epidemiological changes of fracture in children due to home isolation in other countries, and it is beneficial to the decision and treatment of children’s fracture during home isolation.

## Conclusions

Home confinement during the COVID-19 outbreak resulted in a significant reduction in the number of pediatric fractures, especially in male children; however, the number of patients under 4 years old was still considerable, the proportion of 2 to 4 years old patients in 2020 even higher than 2017–2019. Therefore, infants, and pre-school children become the key population of preventing fractures. As the home confinement, the number of patients who need operation treatment decreased. The effect of home isolation on the fracture site and etiology was unnoticeable, the most common site of fractures in children was the upper limb and falling was still the most common cause. Although the number of children with fractures had declined, parents should not relax their care of children during home isolation or after.

## Data Availability

Data will be made available upon requesting the corresponding author on reasonable request.

## Abbreviations

COVID-19: Coronavirus disease 2019
aBMD: Areal bone mineral density

## References

[1] Chen W, Lv H, Liu S, Liu B, Zhu Y, Chen X, Yang G, Liu L, Zhang T, Wang H, Yin B, Guo J, Zhang X, Li Y, Smith D, Hu P, Sun J, Zhang Y (2017). National incidence of traumatic fractures in China: a retrospective survey of 512 187 individuals. Lancet Glob Health.

[2] Rennie L, Court-Brown CM, Mok JYQ, Beattie TF (2007). The epidemiology of fractures in children. Injury.

[3] Schnitzer PG, Dowd MD, Kruse RL, Morrongiello BA (2015). Supervision and risk of unintentional injury in young children. Inj Prev.

[4] Liu JJ, Bao Y, Huang X, Shi J, Lu L (2020). Mental health considerations for children quarantined because of COVID-19. Lancet Child Adolesc Health.

[5] Chen S, Yang J, Yang W, Wang C, Bärnighausen T (2020). COVID-19 control in China during mass population movements at new year. Lancet.

[6] Randsborg P, Gulbrandsen P, Benth JŠ (2013). Fractures in children: epidemiology and activity-specific fracture rates. JBJS..

[7] HedstreM EM, Svensson O, BergstreM U, Michno P (2010). Epidemiology of fractures in children and adolescents. Acta Orthop.

[8] Morrongiello BA, Schell SL, Keleher B (2013). Advancing our understanding of sibling supervision and injury risk for young children. Soc Sci Med.

[9] Bram JT, Johnson MA, Magee LC, Mehta NN, Fazal FZ, Baldwin KD, Riley J, Shah AS (2020). Where have all the fractures gone? The epidemiology of pediatric fractures during the CoVID-19 pandemic. J Pediatr Orthop.

[10] Tore C (2016). Luai, et al. fracture incidence rates in Norwegian children, the Tromsø study, fit futures. Arch Osteoporos.

[11] Wang H, Yu H, Zhou Y (2017). Traumatic fractures as a result of falls in children and adolescents: a retrospective observational study. Medicine (Abingdon).

[12] Naranje SM, Erali RA, Warner WC, Sawyer JR, Kelly DM (2016). Epidemiology of pediatric fractures presenting to emergency departments in the United States. J Pediatr Orthop.

[13] Randsborg P, Gulbrandsen P, altyt Benth J (2013). Fractures in Children: Epidemiology and Activity-Specific Fracture Rates. J Bone Joint Surg.

[14] Rundle AG, Park Y, Herbstman JB, Kinsey EW, Wang YC (2020). COVID-19 Related School Closings and Risk of Weight Gain Among Children. Obesity (Silver Spring).

[15] Dimitri P, Wales JK, Bishop N (2010). Fat and bone in children: differential effects of obesity on bone size and mass according to fracture history. J Bone Miner Res.

[16] Strong WB, Malina RM, Blimkie CJR, Daniels SR, Dishman RK, Gutin B, Hergenroeder AC, Must A, Nixon PA, Pivarnik JM, Rowland T, Trost S, Trudeau F (2005). Evidence based physical activity for school-age youth. J Pediatr.

[17] Bailey DA, McKay HA, Mirwald RL, Crocker P, Faulkner RA (1999). A six-year longitudinal study of the relationship of physical activity to bone mineral accrual in growing children: the university of Saskatchewan bone mineral accrual study. J Bone Miner Res.

